# Conditional disease-free survival in high-risk renal cell carcinoma treated with sunitinib

**DOI:** 10.18632/aging.102549

**Published:** 2019-12-11

**Authors:** Ning Shao, Hengchuan Su, Dingwei Ye

**Affiliations:** 1 Department of Urology, Fudan University Shanghai Cancer Center, Shanghai 200032, China; 2 Department of Oncology, Shanghai Medical College, Fudan University, Shanghai 200032, China

**Keywords:** conditional disease-free survival, high-risk, renal cell carcinoma, sunitinib, patient counseling

## Abstract

Background: Disease-free survival (DFS) did not reflect accurate individual prognosis after initial diagnosis. As conditional DFS (CDFS) could provide dynamic prognostic information, we evaluated CDFS in these patients treated with or without sunitinib.

Results: A total of 1329 patients with median follow-up 6.54 years were enrolled. CDFS improved continuously with disease-free survivorship increasing in both sunitinib and placebo group with minimal difference. In placebo arm, the CDFS of surviving to five year after living 1, 2, 3, and 4 years were 65%, 78%, 87%, and 95% (observed 5-year DFS: 51%). Dynamic changes of HR showed adjuvant sunitinib decrease relapse risks during the first 1.5 years after surgery (P < 0.03).

Conclusions: Our study provided contemporary data of CDFS and change of relapse HR in high-risk ccRCC patients after adjuvant sunitinib or placebo. The remarkable improvement in CDFS highlighted the importance of disease-free interval as a strong indicator in patient counseling and surveillance planning.

Materials and Methods: The primary end point was CDFS and the second end point was smooth hazard ratios (HR) for the prediction of relapses. The differences of conditional survival were compared with the calculation of d value.

## INTRODUCTION

Approximately 70% of renal cell carcinoma (RCC) patients are diagnosed without metastatic disease. One fifth of these patients will develop metastatic RCC (mRCC) after initial nephrectomy [1]. Since approved in mRCC, adjuvant tyrosine kinase inhibitors (TKIs) were attempted in high-risk patients in several trials [2]. In light of the disease-free survival (DFS) benefit with sunitinib, the National Comprehensive Cancer Network (NCCN) guideline provides three options for high-risk clear cell RCC (ccRCC) patients, including clinical trials, surveillance and adjuvant sunitinib [3].

However, DFS did not reflect accurate individual prognosis after initial diagnosis. Measures of prognosis become less relevant as the time from diagnosis increases. Nevertheless, conditional survival (CS), came from the conception of conditional probability, may provide more applicable individual prognosis than DFS at each follow-up time [2, 4]. CS is a dynamic parameter of cumulative survival from follow-up time points on the basis of the condition of survivorship, which is different from traditional survival. CS and its usefulness had been proven in several solid malignancies [5, 6]. Previous CS analyses in high-risk ccRCC patients after surgery didn’t provide information regarding DFS. Therefore, the purpose of this study was to evaluate conditional DFS (CDFS) in high-risk ccRCC treated with or without adjuvant sunitinib based on two large RCTs (S-TRAC and ASSURE) [7, 8]. These CDFS have meaningful implications for clinical counseling and surveillance planning.

## RESULTS

**Figure 1:**
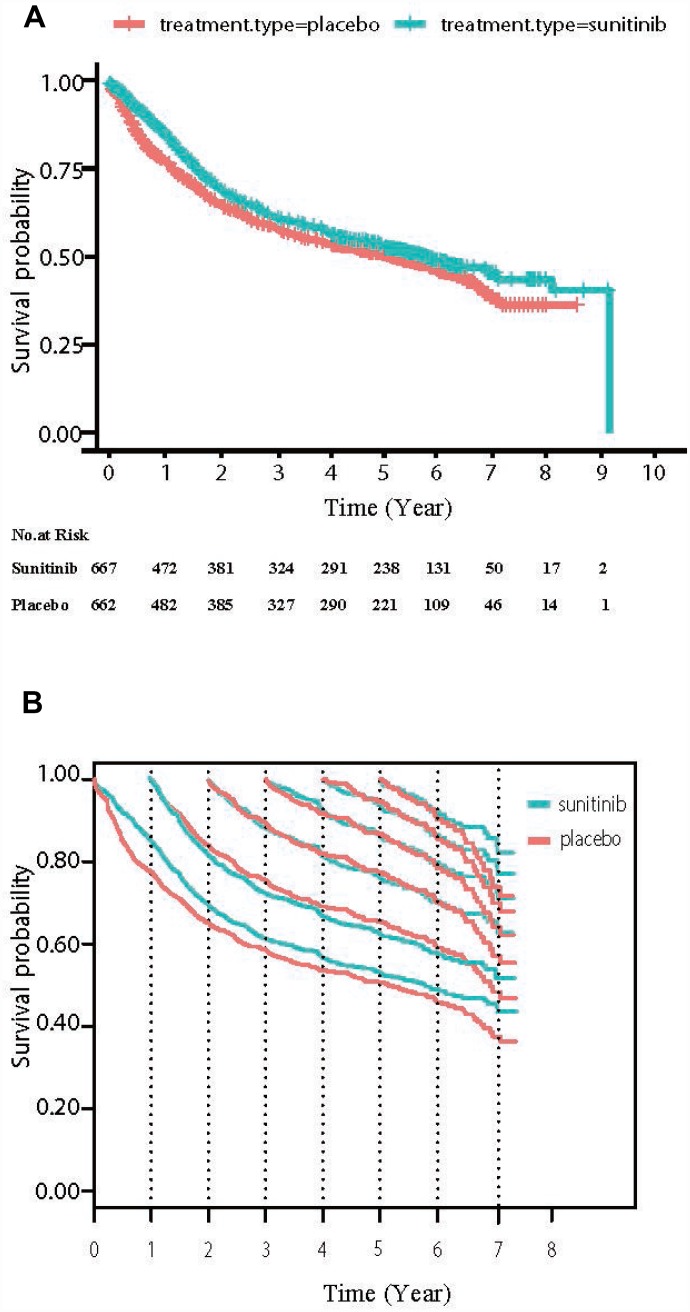
(**A**) The overall DFS curve of high-risk (T3/4 or N1) ccRCC patients treated with adjuvant sunitinib or placebo. (**B**) Conditional DFS (CDFS) curves according to the number of years after randomization. Traditional Kaplan-Meier estimates of DFS (the starting point of the X axis = 0) overlaid by conditional DFS estimates at 1yr (the starting point of the X axis = 1), 2yr (the starting point of the X axis = 2), 3yr (the starting point of the X axis = 3) and so on are shown from the time of randomization.

A total of 1329 patients (sunitinib group n = 667; placebo group n = 662) were enrolled for CDFS analysis. Of these patients, 948 (71.33%) patients were male and 1177 (88.56%) were from majority (white) populations (Supplementary Table 2). With a median follow-up of 6.54 years, the 3 and 5 years DFS were 61% and 53% for sunitinib, while 59% and 51% for placebo. DFS did not differ significantly between the groups (HR = 0.86, 95% CI= 0.73-1.00, *P* = 0.05, Figure 1A).

Table 1 shows the CDFS at different time points for high-risk ccRCC patients treated with adjuvant sunitinib or placebo. CDFS improved continuously with disease-free survivorship increasing in both sunitinib and placebo group. In placebo group, the CDFS of surviving to five year after living 1, 2, 3, and 4 years were 65%, 78%, 87%, and 95%, respectively (much higher than the observed 5-year DFS: 51%). In addition, the CDFS were almost the same in the two groups. The 3-year CDFS after having treated with sunitinib for 1, 2 and 3 year was 66%, 77% and 80%, respectively, compared to 69%, 78% and 78% in the placebo group (very small difference, d value was <0.06, Figure 1B). Additionally, the COS also yielded similar results in the two groups (Table 2 and Supplementary Figure 2). The 3-year COS after having treated with sunitinib for 1, 2 and 3 year was 86%, 86% and 85%, respectively, compared to 86%, 86% and 84% in the placebo group (very small difference).

**Table 1:** Conditional DFS at various time points.

**Time point by study type**	**Conditional DFS since time point (months)**														
	**Observed survival**			**12**		**24**		**36**		**48**		**60**		**72**	
	**%**	**95% CI**	**No.at Risk**	**%**	**95% CI**	**%**	**95% CI**	**%**	**95% CI**	**%**	**95% CI**	**%**	**95% CI**	**%**	**95% CI**
Placebo(months)															
3	93	91-95	606	79	76-83	68	64-72	61	57-65	57	53-61	54	49-58	49	44-53
6	83	80-86	531	83	80-86	72	68-76	65	61-69	62	57-66	57	52-61	50	45-56
12	78	74-81	483	84	81-87	75	71-79	69	65-73	65	61-70	59	54-64	48	41-55
18	71	67-75	432	86	83-90	78	74-82	74	70-78	68	64-73	60	55-66	51	43-59
24	65	61-69	386	90	87-93	82	78-86	78	74-82	70	65-76	58	50-66	56	47-64
36	59	55-63	328	92	89-95	87	83-91	78	73-84	64	55-73	62	53-71		
48	54	50-58	291	95	92-97	86	81-91	70	61-79	68	58-78				
Sunitinib(months)															
3	96	95-98	624	84	81-87	70	66-73	63	59-67	58	53-62	54	50-58	50	45-54
6	92	90-94	577	84	81-87	71	67-75	65	60-69	59	55-64	56	51-60	51	46-56
12	85	82-88	495	82	78-85	72	68-76	66	62-71	62	58-67	58	53-62	53	48-59
18	77	74-81	437	84	81-88	77	73-81	71	66-75	66	62-71	61	56-66	57	50-63
24	69	66-73	381	88	85-92	82	78-86	77	72-81	71	66-76	66	60-72	63	56-70
36	61	57-65	325	92	90-95	87	83-91	80	75-85	74	68-80	71	64-78		
48	57	53-61	292	94	91-97	86	82-91	80	74-87	77	70-84				

**Table 2:** Conditional OS at various time point.

**Time Point by Study Type**	**Conditional OS since Time Point (months)**														
		**Observed Survival**		**12**		**24**		**36**		**48**		**60**		**72**	
	**%**	**95% CI**	**No.at Risk**	**%**	**95% CI**	**%**	**95% CI**	**%**	**95% CI**	**%**	**95% CI**	**%**	**95% CI**	**%**	**95% CI**
Placebo(months)															
6	99	99-100	634	95	93-97	91	89-93	87	85-90	82	79-85	77	73-80	73	70-77
12	98	97-99	612	95	93-96	91	89-93	86	84-89	81	78-84	76	72-80	72	68-76
18	95	94-97	590	96	94-97	92	89-94	86	83-89	81	77-84	77	73-81	72	67-77
24	93	91-95	569	96	94-98	91	89-94	86	83-89	80	77-84	76	72-80	69	62-76
36	89	86-91	523	95	93-97	89	87-92	84	80-87	79	75-83	72	65-79		
48	84	81-87	483	94	92-96	88	85-91	76	68-83	66	54-78				
Sunitinib(months)															
6	99	98-100	627	96	94-97	91	89-93	86	83-89	86	79-86	79	76-82	74	70-78
12	97	96-98	588	95	93-96	91	88-93	86	83-89	82	78-85	77	74-81	72	68-76
18	94	93-96	568	95	93-97	90	88-92	86	83-89	83	79-86	77	73-81	69	64-74
24	92	90-94	544	96	94-98	91	88-93	86	83-89	82	78-85	76	72-80	68	63-74
36	88	85-91	502	95	93-97	90	87-93	85	82-89	79	75-83	71	66-77		
48	84	81-87	457	95	93-97	90	87-93	83	80-87	75	70-81				

**Figure 2:**
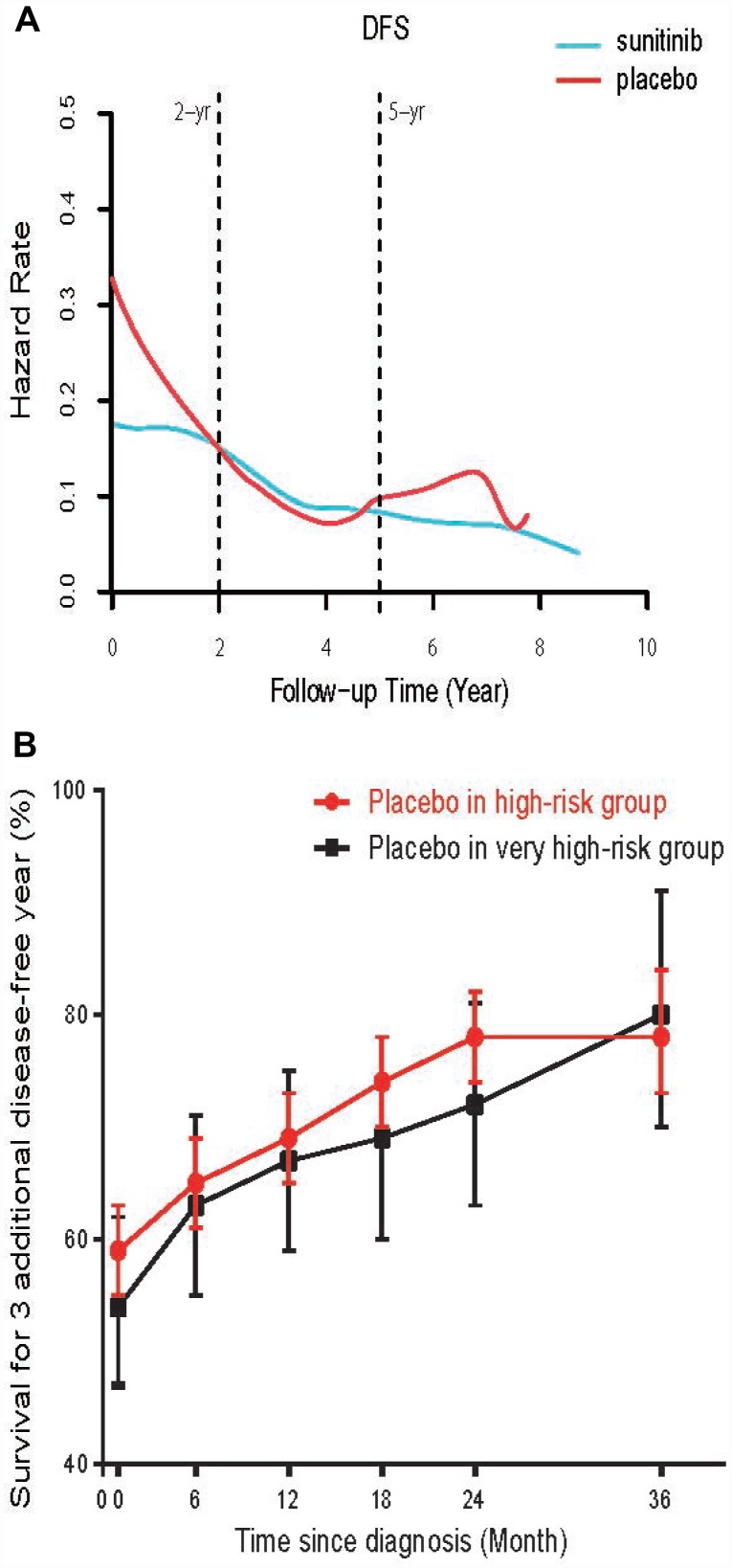
(**A**) The smooth estimate of HR for relapse among high-risk ccRCC patients treated with adjuvant sunitinib or placebo. (**B**) Conditional probability of surviving an additional 3 disease-free year at various time points in very high-risk (T3, Fuhrman grade 2, ECOG Performance Status≥1; or T4 or N1) and high-risk group. The dots represent the probability point estimates, and the vertical bars represent the 95% CIs of the corresponding point estimates.

Figure 2A showed a smooth estimate of HR of disease relapse in high-risk ccRCC patients with adjuvant sunitinib or placebo. In both group, the likelihood of relapse was not uniform over time but peaked at the beginning and diminished onwards. Sunitinib decreased the risk of relapse compared with placebo in the first 1.5 years (HR at 0.5, 1 and 1.5 years was 0.171, 0.172 and 0.166, vs. placebo 0.267, 0.225 and 0.185, respectively, all P < 0.03). After 1.5 years, no significant difference was observed between two groups (P>0.05).

We then tested the trend of CDFS change in patients with very high-risk of recurrence. In placebo group, the CDFS of these patients surviving to five year after surviving 1, 2, 3, and 4 years improved from observed 5-year DFS 47% to 62%, 72%, 87% and 93%, respectively. Although the CDFS in very high-risk patients were lower than that of high-risk patients at the beginning, the increase of CDFS were more prominent in very high-risk subset after living disease free for 3 years (Figure 2B, Supplementary Table 3). Additionally, we also test whether adjuvant sunitinib dose may alter CDFS during follow-up. Using high-risk patients from ASSURE, those treated with high dose (≥ 1246 mg/cycle) sunitinib did not have a better CDFS significantly than these patients treated with low dose (<828 mg/cycle, small differences, d value was <0.3, Supplementary Table 3).

## DISCUSSION

Tumor is a complex systemic disease [9, 10]. Most RCC patients have more than 5 years life expectancies after diagnosis. Therefore, traditional survival estimates are inappropriate to patients who have survived a period of time after initial diagnosis and treatment [11, 12]. It was previously proved that the risk of death from the cancer decreases with increasing length of survival [13, 14]. A question, such as ”Now that I’m 2 years out from adjuvant treatment, what’s my expected survival for another 5 years?”, may be posed by patients. At this juncture, physicians usually have little guidance on these patients due to few clinical data. The problem is even more prominent in ccRCC after surgery, because nearly half of high-risk (T3/4 or N1) patients were disease-free after 4-year follow-up [15].

Previous studies evaluated CS in surgical treated ccRCC. Most studies focused on overall survival, which may be influenced by post-recurrence treatment [2]. Even for these important RCTs, few could provide CS data. We analyzed the OS data and found a high survival rate for both groups. The change of COS was very small within the median follow-up of 6.54 years and provided little information of the “cure” probability after surgery. In addition, most novel agents were approved duo to their improvement on DFS. Hence, the CDFS was the primary end point of our study. We assessed CDFS in high-risk ccRCC patients after surgery and adjuvant sunitinib or placebo. It was a large sample retrospective analysis and the data were interesting. The results suggested that the estimated 1-year DFS rate for patients who had lived for 3 years without relapse may be higher than that of patients recently diagnosed. This also indicated that CS could provide dynamic and personalized prognostic information, which was important guide subsequent follow-up plan. Previous studies also confirmed the advantages of CS, some of which had provided the CS data after nephrectomy for RCC [16]. However, few focused on the CS of patients treated with or without adjuvant sunitinib, especially for CDFS. Hence, our study provided significant prognostic information in this field.

The analyses of two large prospective trials give convincible and contemporary data for two optional choices according to guidelines. Patients and physicians could obtain CS information they are interested in from our study. There are several points to note from this study. First, CDFS improved continuously with disease-free survivorship increasing in high-risk ccRCC patients. The unanticipated good outcome highlighted the importance of accurate risk reclassification during the follow-up. Second, the increase of CDFS was more prominent in very high-risk subset according to UISS classification after living disease free for 3 years. The 3-year CDFS was 80% after disease-free for 3 years, which give them a comfort and extra confidence to fight with diseases. Third, we found a reduction of disease failure only during the first 1.5 years in the sunitinib group based on our analysis of HR changes. NCCN guideline indicated that the median time to relapse after initial surgery was 1 to 2 years, with most relapse occurring within 3 years. In our analysis, 76.9% of relapses in high-risk patients occurred within 3 years after surgery. These observations suggested: Sunitinib prevent occult metastases growth shortly after surgery. Since in metastatic RCC patients treated with sunitinib the median PFS was 9.5 months. Therefore, adjuvant sunitinib may only control occult metastatic disease for a short peroid and tumor still progressed due to refractory. This may explain the lack of significant differences in recurrence risk between two groups for long term survivors. The risk of recurrence was almost plateau from 3 to 7 years, which suggesting a long term follow-up is still mandatory. Recently, evidence from adjuvant therapy for melanoma showed target therapy reduced recurrence immediately in the short peroid after surgery, while immunotherapy reduced recurrence on a later time [17]. Therefore, a combination of anti-angiogenesis and immunotherapy may have widespread prospects in high-risk ccRCC treatment and warrant further evaluation in the adjuvant setting.

Considering more accurate survival information of CDFS for long-term prognosis than DFS, this study could be conducive to patient counseling, surveillance planning, adjuvant therapy decisions and design of future clinical trials.

The present study also has some limitations. The present study was based on survival plot in published articles. Therefore, the grouping of patients was pre-specified according to UISS classification and a comprehensive subgroup analyses could not be done. Since only 0.08% of enrolled patients had PS≥2, the data from randomized trials may not generalize to patients in community setting. Additionally, most of the included patients were white people. Ethnicity issue should be considered in future studies.

## CONCLUSIONS

In conclusion, our study provided contemporary data of CDFS and change of relapse HR in high-risk ccRCC patients after adjuvant sunitinib or placebo. The remarkable improvement in CDFS during follow-up highlighted the importance of disease-free interval as a strong indicator in patient counseling and surveillance planning.

## METHODS

### Patients

High-risk (T3/4 or N1) ccRCC patients from S-TRAC and ASSURE, who were randomized to receive adjuvant sunitinib or placebo up to 1 year, were included. The eligible criteria, treatment approaches, definition of outcome and follow-up were compared between S-TRAC and ASSURE (Supplementary Table 1) [15, 18]. Individual patient data of DFS and overall survival (OS) were digitally reconstructed from S-TRAC and ASSURE studies using R and DigitizeIt software (Supplementary Figure 1). Subgroup analysis of very high-risk (defined as T3/T4, or node positive disease, no metastasis, Fuhrman grade 2 or higher and an ECOG score of 1 or higher) from S-TRAC and different dose received from ASSURE were also performed.

Previous studies described the steps to digitally reconstruct patient-level data on time-to-event outcome and treatment and biomarker groups using published Kaplan-Meier survival curves [19]. The reconstructed dataset and the corresponding computer programs are publicly available to enable further statistical methodology research. The methods were widely used in researches of JAMA oncology or Lancet [20, 21]. We used the method and the available R code to obtain individual patient data. Each data includes individual treatment type and possibly censored time to event data consistent with a published Kaplan-Meier curve.

### Statistical analysis

The primary end point in our study was CDFS and second end points included conditional overall survival (COS) and hazard ratios (HR) changes over the follow-up course. CS is the proportion surviving. CDFS or COS can be estimated from individual patient data using the multiplicative law of probability. For instance, 3 additional years, per the following equation: when S(x) is overall survival at time x, conditional survival is S(x +3)/S(x). Standardized differences (d) were calculated to assess the differences of CS between subgroups using the method introduced by Cucchetti et al [5, 22]. The standardized difference in proportions is measured by (P2 – P1)/√ [P(1-P)] where P is the weighted mean of P1 and P2. Smoothed HR was evaluated and plotted using “muhaz” R package, which is producing a smooth estimate of the hazard function from censored data using kernel-based methods. P-value was calculated to estimate the differences of CS between different groups. It was calculated by bootstrap test using the difference in smoothed HR as the test statistic. Specifically, the samples were pooled, two groups of samples of the original group sizes were resampled with replacement from the pooled data and the test statistic was re-calculated. The process was repeated 1,000 times and the p-value was calculated as the percentage of bootstrap samples that have a test statistic more extreme than the observed test statistic.

## Supplementary Material

Supplementary Materials

Supplementary Figures

Supplementary Tables
