# Supplementary material for: Conditional disease-free survival in high-risk renal cell carcinoma treated with sunitinib
Source: Aging (Albany NY). 2019 Dec 11;11(23):11490–503. doi: 10.18632/aging.102549 (PMC6932878; doi:10.18632/aging.102549)
Supplement: Supplementary Materials [file aging-11-102549-s001..pdf]

SUPPLEMENTARY METHODS

Additional details on statistical analyses

Disease-free survival (DFS) defined as the time from randomization to recurrence, development of second primary cancer, or death from any cause. Overall survival (OS) defined as measured from date of randomization to date of death, from any cause. Conditional survival (CS) measures the probability that a cancer patient will survive some additional number of years, given that the patient has already survived for a certain number of years.

Retrieve individual patient data

The data presented in this article were digitally reconstructed from figures published in S-TRAC and ASSURE studies using R and DigitizeIt software. [1, 2]

The updated S-TRAC results only provided the subgroup (T3, no or undetermined nodal involvement, no metastasis, Fuhrman grade ≥2, ECOG PS >1;or T4 and/or nodal involvement) analyses for DFS, but all high-risk patients (T3 or T4, or node positive disease or any T stage with local nodal involvement) for OS analysis. [3] Furthermore, the updated ASSURE results only included high-risk patients (pT3, pT4, or node positive disease). [4] Therefore, to evaluate conditional DFS (CDFS) in high-risk localized RCC, DFS data of ASSURE reconstructed from the updated study published in 2018. DFS data of S-TRAC used the study published in 2016. OS data of S-TRAC and ASSURE reconstructed from both updated study.

Previous studies described the steps to digitally reconstruct patient-level data on time-to-event outcome and treatment and biomarker groups using published Kaplan-Meier survival curves. [5] The reconstructed dataset and the corresponding computer programs are publicly available to enable further statistical methodology research. The methods were widely used in researches of JAMA oncology or Lancet. [6, 7] We used the method and the available R code to obtain individual patient data (Supplementary Figure 4). Each data includes individual treatment type and possibly censored time to event data consistent with a published Kaplan-Meier curve.

Generate CS results

Conditional survival is the proportion surviving. For example, 3 additional years, per the following equation: when S(t) is overall survival at time t, conditional survival is S(x +3)/S(x). Standardized differences (d) were used to assess the differences of CS between subgroups based on the method described by Cucchetti et al. [8, 9] The standardized difference in proportions is calculated as $(P2 - P1)/\sqrt{[P(1-P)]}$ where P is the weighted mean of P1 and P2:

1) d values lower than |0.1| indicate very small differences between means; 2) d values between |0.1| and |0.3| indicate small differences; 3) d values between |0.3| and |0.5| indicate moderate differences; 4) and d values greater than |0.5| indicate considerable differences.

Generate smoothed HR

Smoothed HR was evaluated and plotted using “muhaz” R package, which is producing a smooth estimate of the hazard function from censored data using kernel-based methods. P-value was used to assess the differences of CS between different groups. It was calculated by bootstrap test using the difference in smoothed HR as the test statistic. Specifically, the samples were pooled, two groups of samples of the original group sizes were resampled with replacement from the pooled data and the test statistic was re-calculated. The process was repeated 1,000 times and the p-value was calculated as the percentage of bootstrap samples that have a test statistic more extreme than the observed test statistic.

REFERENCES

1. Ravaud A, Motzer RJ, Pandha HS, et al. Adjuvant Sunitinib in High-Risk Renal-Cell Carcinoma after Nephrectomy. The New England journal of medicine. 2016;375:2246–54. https://doi.org/10.1056/nejmoa1611406 PMID:27718781

2. Haas NB, Manola J, Uzzo RG, et al. Adjuvant sunitinib or sorafenib for high-risk, non-metastatic renal-cell carcinoma (ECOG-ACRIN E2805): a double-blind, placebo-controlled, randomised, phase 3 trial. Lancet. 2016;387:2008–16. https://doi.org/10.1016/s0140-6736(16)00559-6

3. Motzer RJ, Ravaud A, Patard JJ, et al. Adjuvant Sunitinib for High-risk Renal Cell Carcinoma After Nephrectomy: Subgroup Analyses and Updated Overall Survival Results. European urology. 2018;73:62–8. https://doi.org/10.1016/j.eururo.2017.10.021 PMID: 28967554

4. Haas NB, Manola J, Dutcher JP, et al. Adjuvant Treatment for High-Risk Clear Cell Renal Cancer: Updated Results of a High-Risk Subset of the ASSURE

Randomized Trial. JAMA oncology. 2017;3:1249–52. https://doi.org/10.1001/jamaoncol.2017.0076
PMID: 28278333

5. Satagopan JM, Iasonos A, Kanik JG. A reconstructed melanoma data set for evaluating differential treatment benefit according to biomarker subgroups. Data in brief. 2017;12:667–75. https://doi.org/10.1016/j.dib.2017.05.005
PMID: 28560273

6. Aguiar PN, Jr., Haaland B, Park W, San Tan P, Del Giglio A, de Lima Lopes G, Jr. Cost-effectiveness of Osimertinib in the First-Line Treatment of Patients With EGFR-Mutated Advanced Non-Small Cell Lung Cancer. JAMA oncology. 2018;4:1080–4. https://doi.org/10.1001/jamaoncol.2018.1395
PMID: 29852038

7. Chu DK, Kim LH, Young PJ, et al. Mortality and morbidity in acutely ill adults treated with liberal versus conservative oxygen therapy (IOTA): a systematic review and meta-analysis. Lancet. 2018;391:1693–705. https://doi.org/10.1016/s0140-6736(18)30479-3
PMID:27718781

8. Cucchetti A, Piscaglia F, Cescon M, et al. Conditional survival after hepatic resection for hepatocellular carcinoma in cirrhotic patients. Clinical cancer research : an official journal of the American Association for Cancer Research. 2012;18(16):4397-405. https://doi.org/10.1158/1078-0432.CCR-11-2663
PMID:22745107

9. Kim Y, Margonis GA, Prescott JD, et al. Curative Surgical Resection of Adrenocortical Carcinoma: Determining Long-term Outcome Based on Conditional Disease-free Probability. Annals of surgery. 2017;265(1):197-204. https://doi.org/10.1097/SLA.0000000000001527
PMID:28009746
