# Supplementary material for: Conditional disease-free survival in high-risk renal cell carcinoma treated with sunitinib
Source: Aging (Albany NY). 2019 Dec 11;11(23):11490–503. doi: 10.18632/aging.102549 (PMC6932878; doi:10.18632/aging.102549)
Supplement: Supplementary Figures [file aging-11-102549-s003..pdf]

SUPPLEMENTARY FIGURES

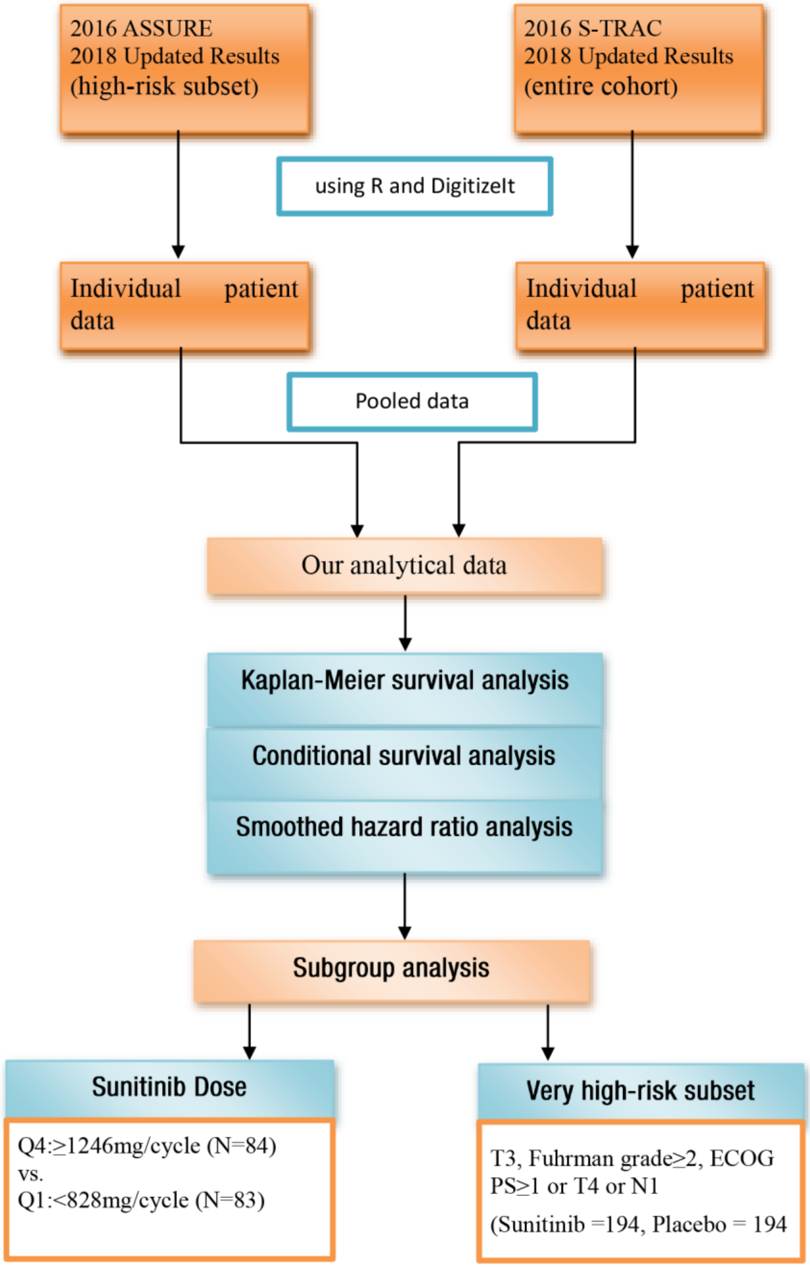

Supplementary Figure 1. A flow chart to obtain analytical data.

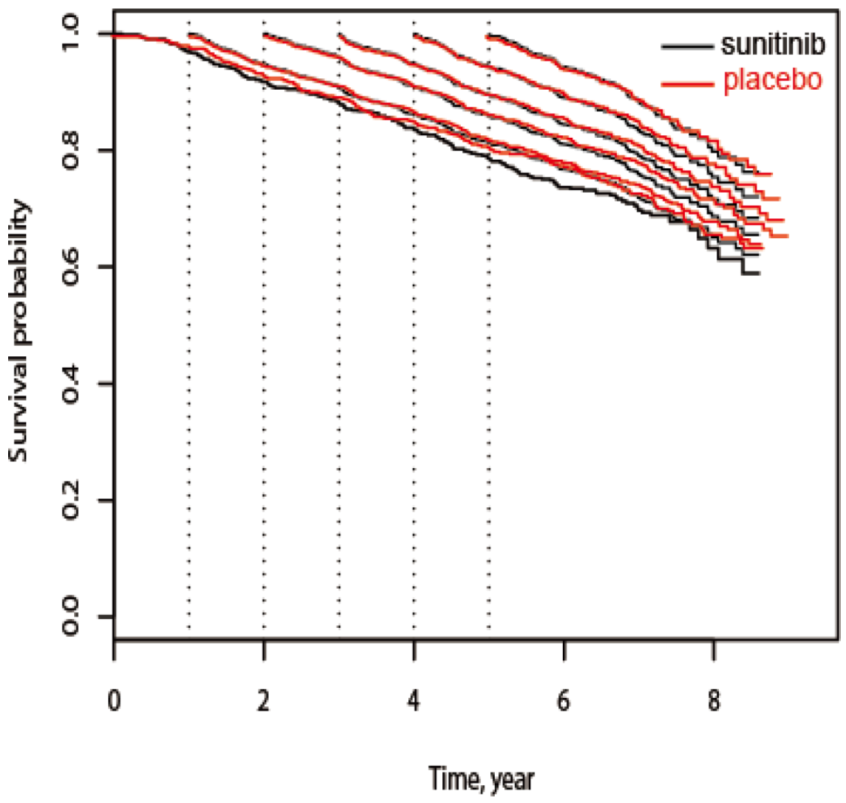

Supplementary Figure 2. Conditional DFS curves according to the number of years from treatment. Conditional survival (CS) curves according to the number of years after randomization. Traditional Kaplan-Meier estimates of CS (the starting point of the X axis = 0) overlaid by conditional CS estimates at 1yr (the starting point of the X axis = 1), 2yr (the starting point of the X axis = 2), 3yr (the starting point of the X axis = 3) and so on are shown from the time of randomization.
