# Supplementary material for: Conditional disease-free survival in high-risk renal cell carcinoma treated with sunitinib
Source: Aging (Albany NY). 2019 Dec 11;11(23):11490–503. doi: 10.18632/aging.102549 (PMC6932878; doi:10.18632/aging.102549)
Supplement: Supplementary Tables [file aging-11-102549-s002..pdf]

SUPPLEMENTARY TABLES

Supplementary Table 1. Basic characteristic of S-TRAC and ASSURE.

	S-TRAC	ASSURE (High-risk subset)
Time<br>Geography<br>Eligible criteria	2007-2011<br>multinational<br>ccRCC	2006-2010<br>United states and Canada<br>ccRCC
	T3/4N0M0 or TxN1M0<br>no previous systemic treatment	T3/4N0M0 or TxN1M0
	treatment initiation within 3 to 12 weeks after nephrectomy	within 12 weeks of remova of the primary tumor
Primary end point	disease-free survival	disease-free survival
Treatment	sunitinib (50 mg/day) or placebo on a 4-weeks-on, 2-weeks-off	sunitinib (50 mg/day) or placebo per day for 4 weeks of every 6 weeks
		Amended of sunitinb to 37.5mg with mandatory dose escalation if no serous adverse effects
		Allowed sunitinib dosing as low as 25mg/day
Follow-up	every 12 weeks during the first 3 years and every 6 months year 4 to 5, every 12 months thereafter	every three cycles (4·5 months) during year 1, then every 6 months for year 2, and then once per year for 10 years during follow-up.

Supplementary Table 2. Baseline characteristics of the patients.

	Patients, n (%)					
	Sunitinib N=667		Placebo N=662		Whole N=1329	
Gender no. (%)						
Male	465	69.70%	483	72.96%	948	71.33%
Female	202	30.30%	179	27.04%	381	28.67%
Race no. (%)						
White	593	88.90%	584	88.22%	1177	88.56%
Black	12	1.80%	14	2.12%	26	1.96%
Asian	45	6.75%	44	6.65%	89	6.70%
Other	12	1.80%	12	1.80%	24	1.80%
Unknown	5	0.75%	8	1.21%	13	0.98%
ECOG Performance Status no. (%)						
0	502	75.26%	499	75.38%	1001	75.31%
1	163	24.44%	161	24.32%	324	24.37%
≥2	1	0.15%	0	0	1	0.08%
Unknown	1	0.15%	2	0.30%	3	0.24%
Disease Stage no. (%)						
T3N0/xM0	610	91.5%	593	89.6%	1203	90.5%
T4N0/xM0	9	1.3%	5	0.8%	14	1.1%
TxN1M0	48	7.2%	64	9.7%	112	8.4%

Supplementary Table 3. Subgroup conditional DFS at various time point.

Time Point by Study Type	Observed Survival			Conditional DFS since Time Point (months)									
				12		24		36		48		60	
	%	95% CI	No.at Risk	%	95% CI	%	95% CI	%	95% CI	%	95% CI	%	95% CI
High dose Sunitinib (month)													
6	84	80-86	84	83	74-91	69	59-80	60	48-71	58	47-69	55	44-67
12	77	74-81	67	86	78-95	74	64-85	65	53-77	63	52-75	57	45-70
18	73	67-75	60	84	74-93	72	61-83	70	59-82	67	55-79	59	46-72
24	66	61-69	57	86	77-95	75	64-86	73	62-85	66	53-79	52	36-68
36	60	55-63	48	87	78-97	85	75-95	77	64-90	61	43-78	61	43-78
48	55	50-58	39	97	92-100	88	77-99	69	51-88	69	51-88	69	51-88
Low dose Sunitinib (month)													
6	92	90-94	83	83	74-92	66	54-78	59	46-71	51	38-64	46	32-59
12	85	82-88	59	77	66-88	63	51-76	61	48-74	52	38-66	47	32-61
18	77	74-81	47	80	68-91	71	58-84	61	47-75	55	40-70	48	32-64
24	69	66-73	41	82	71-94	80	67-92	68	53-83	60	44-77	56	39-74
36	61	57-65	31	97	91-100	82	68-96	73	56-91	68	49-87	61	40-82
48	57	53-61	29	85	71-99	76	59-93	70	51-89	63	42-85		
Very high-risk Sunitinib (month)													
6	82	80-86	194	86	80-91	74	67-81	69	62-77	65	57-73	60	52-69
12	77	74-81	143	82	75-88	75	68-82	70	63-78	68	60-76	62	53-71
18	70	67-75	123	86	80-92	81	74-88	75	68-83	70	62-79	64	54-74
24	64	61-69	109	92	86-97	86	79-93	83	76-90	76	67-85	67	55-80
36	57	55-63	98	94	89-99	90	84-96	83	74-91	73	60-87		
48	54	50-58	89	96	92-100	88	80-96	78	65-92				
Very high-risk Placebo (month)													
6	92	90-94	194	84	78-89	70	63-78	63	55-71	58	49-66	55	46-63
12	86	82-88	134	86	80-92	71	64-79	67	59-75	62	54-71	57	48-67
18	76	74-81	123	84	77-91	75	67-83	69	60-78	65	56-75	59	49-70
24	68	66-73	110	83	76-90	78	70-86	72	63-81	66	56-77		
36	62	57-65	83	94	89-99	87	80-95	80	70-91				
48	56	53-61	76	93	87-99	85	76-95						
